# Odontogenic gene expression profile of human dental pulp-derived cells under high glucose influence: a microarray analysis

**DOI:** 10.1590/1678-7757-2020-1074

**Published:** 2021-09-27

**Authors:** Sivaporn HORSOPHONPHONG, Hathaitip SRITANAUDOMCHAI, Siriruk NAKORNCHAI, Nakarin KITKUMTHORN, Rudee SURARIT

**Affiliations:** 1 Mahidol University Faculty of Dentistry Department of Oral Biology Thailand Mahidol University, Faculty of Dentistry, Department of Oral Biology, Thailand.; 2 Mahidol University Faculty of Dentistry Department of Pediatric Dentistry Thailand Mahidol University, Faculty of Dentistry, Department of Pediatric Dentistry, Thailand.

**Keywords:** Microarray analysis, Human dental pulp- derived cells, Mineralization, High glucose, Hyperglycemia

## Abstract

Hyperglycemia, a major characteristic of diabetes, is considered to play a vital role in diabetic complications. High glucose levels have been found to inhibit the mineralization of dental pulp cells. However, gene expression associated with this phenomenon has not yet been reported. This is important for future dental therapeutic application.

**Objective:**

Our study aimed to investigate the effect of high glucose levels on mineralization of human dental pulp-derived cells (hDPCs) and identify the genes involved.

**Methodology:**

hDPCs were cultured in mineralizing medium containing 25 or 5.5 mM D-glucose. On days 1 and 14, RNA was extracted and expression microarray performed. Then, differentially expressed genes (DEGs) were selected for further validation using the reverse transcription quantitative polymerase chain reaction (RT-qPCR) method. Cells were fixed and stained with alizarin red on day 21 to detect the formation of mineralized nodules, which was further quantified by acetic acid extraction.

**Results:**

Comparisons between high-glucose and low-glucose conditions showed that on day 1, there were 72 significantly up-regulated and 75 down-regulated genes in the high-glucose condition. Moreover, 115 significantly up- and 292 down-regulated genes were identified in the high-glucose condition on day 14. DEGs were enriched in different GO terms and pathways, such as biological and cellular processes, metabolic pathways, cytokine–cytokine receptor interaction and AGE-RAGE signaling pathways. RT-qPCR results confirmed the significant expression of pyruvate dehydrogenase kinase 3 (PDK3), cyclin-dependent kinase 8 (CDK8), activating transcription factor 3 (ATF3), fibulin-7 (Fbln-7), hyaluronan synthase 1 (HAS1), interleukin 4 receptor (IL-4R) and apolipoprotein C1 (ApoC1).

**Conclusions:**

The high-glucose condition significantly inhibited the mineralization of hDPCs. DEGs were identified, and interestingly, HAS1 and Fbln-7 genes may be involved in the glucose inhibitory effect on hDPC mineralization.

## Introduction

Diabetes mellitus constitutes a group of metabolic disorders related to hyperglycemia. The adverse effects of diabetes mellitus include damage, destruction and failure of various tissues, including oral and dental tissues.^1^ Dental pulp is one of the tissues affected by diabetes. Studies have reported alteration in dental pulp structure, increased inflammation and impaired pulpal healing in the dental pulp of people with diabetes.^2^^,^^3^ In addition, *in vivo* studies conducted using animal models have shown that the severity of dental caries and pulpal inflammation were considerably higher in diabetic mice than in non-diabetic controls.^4^^,^^5^ Hyperglycemia has been considered to be a major factor involved in the pathogenesis of diabetes, as it induces alterations at the cellular level of the affected tissue. Most of the cellular and molecular mechanisms involving inflammatory responses and overproduction of reactive oxygen species (ROS) causing tissue damage are observed in several diabetic complications.^6^ Furthermore, hyperglycemia has been shown to interfere with the healing and regeneration of hard tissues. High levels of glucose have also been reported to inhibit the osteogenic differentiation and mineral tissue formation.^5^^,^^7^ Dental pulp is one of the tissues with reparative and regenerative potential.^8^ Although pulp cells have been proved to be useful in tissue engineering and regenerative medicine, the hyperglycemic condition can interfere with its regenerative capacity. Research shows that hyperglycemia exerts a negative effect on pulpal healing by inhibiting dentin bridge formation and increasing pulpal inflammation.^9^ Moreover, treating dental pulp cells with high glucose levels was found to inhibit mineralization.^5^ Even though a high level of glucose was reported to inhibit the mineralization of pulp cells, gene expression associated with the influence of glucose has never been explored and may be important as basis for future studies to improve the dental therapeutic application in diabetic patient.

Expression microarray is a high-throughput technique used to analyze gene expression profiling of cells, tissues and diseases. It is a powerful method to investigate a wide range of transcriptomic profiling. This technique provides details on cellular mechanisms and improves the understanding of the biological processes and the signaling pathways that occur at the cellular level.^10^

Few studies have demonstrated the gene expression pattern of human dental pulp-derived cells (hDPCs) during mineralization. However, there is a lack of studies on hDPCs gene expression profiling during mineralization under the influence of different concentrations of glucose. Therefore, we aimed to investigate the effect of high glucose levels on mineralization of human dental pulp-derived cells (hDPCs) and identify the genes involved.

## Methodology

### Isolation and culture of hDPCs

**Figure S1:**
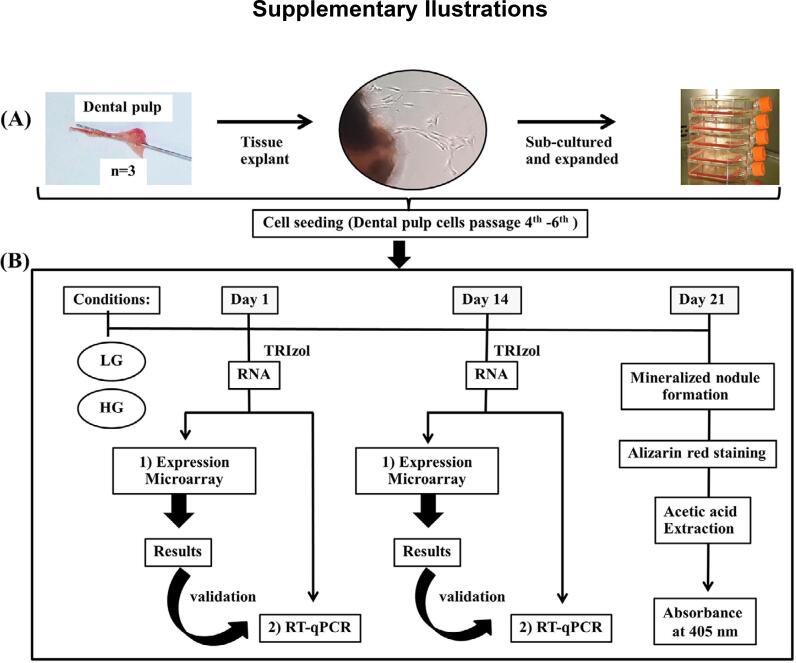
- Experimental flow chart. (A) Isolation of human dental pulp-derived cells using tissue explant technique. (B) Cell mineralization, RNA isolation and analysis of gene expression

This study was approved by the Ethical Committee of the Faculty of Dentistry and Pharmacy, Mahidol University, Thailand (COE: No.MU-DT/PY-IRB 2017/011.3103). Written informed consents were obtained from all donors who participated in the study. Dental pulp tissue was collected from non-carious third molar of 3 healthy donors (2 males and 1 female, aged 18-30 years). hDPCs were isolated using the tissue explant technique as described by Gronthos, et al.^11^ (2011). Briefly, a diamond fissure bur with constant irrigation was used to cut the groove along the cementoenamel junction, after which the tooth was separated, and pulp tissue was collected. The pulp tissue was cut into 0.5–1 mm^2^ fragments, placed in a 60-mm culture dish and then maintained in Dulbecco’s Modified Eagle Medium (DMEM); HyClone, Logan, UT, USA) containing 5.5 mM D-glucose supplemented with 10% fetal bovine serum (FBS; HyClone) and 1% penicillin–streptomycin (Pen-Strep, Gibco BRL, Grand Island, NY, USA), under incubation at 37°C in a humidified atmosphere containing 5% CO_2_. The medium was changed every 3 days. Cells between 4^th^–6^th^ passages were used in this study (Figure S1).

### Experimental design

The hDPCs isolated from 3 individuals were used in this study. These cells were seeded at a density of 1 × 10^5^ cells/well in a 6-well plate and cultured in DMEM containing 10% FBS and supplemented with 50 μg/mL ascorbic acid (Sigma-Aldrich, St. Louis, MO, USA), 10 mM β-glycerophosphate (Sigma-Aldrich) and 100 nM dexamethasone (Sigma-Aldrich) to induce cell mineralization (mineralizing medium; MM). Cells were then incubated under 5% CO_2_ in a humidified atmosphere at 37°C. This was followed by treating with 2 different glucose concentrations, namely 5.5 mM D-glucose (hereinafter, referred to as low glucose; LG) and 25 mM D-glucose (hereinafter, referred to as high glucose; HG). For each sample, the medium was changed every 3 days. RNA isolation was performed on day 1 and day 14. On day 21, the cells were stained with alizarin red S (Sigma-Aldrich) and then quantified for the detection of mineral deposition (Figure S1).

### RNA isolation

After culturing for 1 and 14 days, cells were washed twice with PBS, and RNA was isolated from each sample using TRIzol reagent (Invitrogen, Carlsbad, CA, USA) according to the manufacturer’s protocol. Then, the isolated RNA was treated with DNase I (Thermo Fisher Scientific, Waltham, MA, USA) to remove the contaminating DNA. Concentration, purity and integrity of RNA were evaluated by an ND-1000 Spectrophotometer (NanoDrop, Wilmington, USA) and an Agilent 2100 Bioanalyzer (Agilent Technologies, Palo Alto, USA). The RNA isolated from each sample was divided into two portions; the first portion was used for expression microarray, and the remaining RNA was used for reverse transcription quantitative polymerase chain reaction (RT-qPCR) analysis (Figure S1).

### Expression microarray and data analysis

Gene expression microarray was performed in duplicate using the pooled RNA sample from each individual (n=3). RNA labelling and hybridization were done using the Agilent One-Color Microarray-Based Gene Expression Analysis protocol (Agilent Technologies, V 6.5, 2010). Briefly, 100 ng of total RNA from each sample was amplified and labelled with cyanine 3. Then, the labelled cRNA was fragmented and hybridized using the Agilent SurePrint G3 Human GE 8X60K V3 Microarrays (Agilent Technologies). Raw data were extracted using the software provided by the Agilent Feature Extraction Software (v11.0.1.1). The microarray data are deposited at the Gene Expression Omnibus database (https://www.ncbi.nlm.nih.gov/geo/) under accession number GSE1121144. All data analyses and visualization of differentially expressed genes (DEGs) were conducted using R 3.0.2 (https://www.r-project.org). Statistical significance of the expression data was determined using fold change and local-pooled-error test, where a fold change ≥1.5 and a *q*-value ≤0.05 were considered significantly different in gene expression. Hierarchical cluster analysis was performed using complete linkage and Euclidean distance as a measure of similarity. Gene-enrichment and functional annotation analysis for significant probe list was performed using gene ontology (http://geneontology.org/) and KEGG (https://www.kegg.jp/).

### RT-qPCR

RNA from each sample was used for RT-qPCR validation, wherein 2 μg of the RNA from each sample was reverse-transcribed into cDNA using the RevertAid First Strand cDNA Synthesis Kit (Thermo Fisher Scientific). Real-time PCR was performed using the StepOnePlus Real-Time PCR System (Applied Biosystems, Foster City, CA, USA) using KAPA SYBR qPCR master mix (Kapa Biosystems, Wilmington, MA, USA) for the detection of mRNA expression. The reactions were performed in triplicate using RNA extracted from the 3 different donors. Data were normalized against ubiquitin C (UBC). Relative gene expressions were analyzed using the ΔΔCT method. Mineralization-related genes were also validated. The primer sequences used for the selected genes are described in Table S1. The thermocycling conditions consisted of 95°C for 5 min, followed by 40 cycles of denaturation at 95°C for 15 s, annealing for 30 s (the annealing temperature for each primer is presented in Table S1) and extension at 72°C for 25 s.

### Detection and quantification of mineralized nodule formation

This experiment was conducted to detect the effect of glucose on the mineralization of hDPCs. On day 21, the cells were fixed with 10% (v/v) formaldehyde and stained with 40 mM alizarin red S. The alizarin red S–calcium complex was further quantified as described by Gregory, et al.^12^ (2004). Then, the absorbance was measured at 405 nm in a microplate reader (Synergy H1, Biotek).

### Statistical analysis

Statistical analysis was performed using SPSS version 18 (IBM, Armonk, NY, USA). Independent samples t-test was used for data analysis of RT-qPCR validation and alizarin red S quantification. A p value <0.05 was considered statistically significant.

## Results

### Microarray analysis

**Figure 1:**
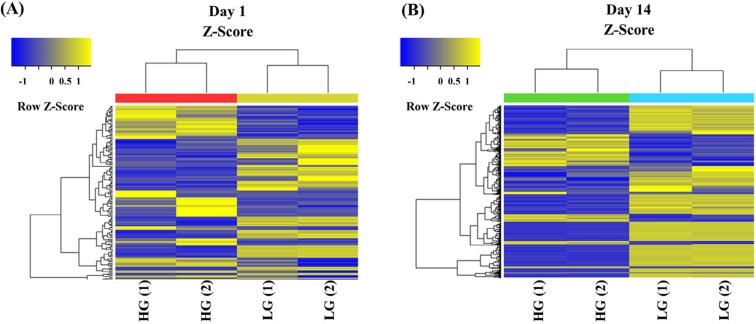
Hierarchical clustering heat map. Hierarchical cluster analysis displayed DEGs compared between low glucose and high glucose on day 1 (A) and day 14 (B). Low glucose (LG), High glucose (HG), sample number 1 (1) and sample number 2 (2)

The number of detected probes was shown in Table S2. Comparisons between high-glucose and low-glucose conditions revealed 72 significantly up-regulated and 75 significantly down-regulated genes in the high-glucose condition on day 1. Moreover, there were 115 significantly up-regulated and 292 down-regulated genes in the high-glucose condition on day 14. Microarray data were analyzed in two pairs of low-glucose and high-glucose conditions on day 1 and day 14. Results of the hierarchical cluster analysis of the DEGs are shown in Figure 1A and B. The top 15 most significantly up- and down-regulated genes are presented in Tables 1 and 2, respectively.

**Table 1:** Top 15 most up-regulated genes under high glucose condition

Day 1				Day 14			
Gene	RefSeq Accession	FC*	q-value	Gene	RefSeq Accession	FC*	q-value
PRDM8	NM_020226	2,02	3,80E-07	TLCD2	NM_001164407	3,46	0
LOC390937	XM_005259448	1,89	0,000291282	MMP1	NM_002421	3,21	0
SSX2	NM_175698	1,84	0,000173783	FBLN7	NM_153214	2,95	5,63E-12
SEMA3F	NM_004186	1,81	4,86E-05	EML1	NM_001008707	2,91	8,98E-10
CLCN6	NM_001256959	1,81	0,000182385	CTC1	NM_025099	2,87	9,36E-10
PCSK1N	NM_013271	1,7	0	SH3BP1	NM_018957	2,74	3,66E-08
FBXO36	NM_174899	1,65	0,001142307	EXPH5	NM_015065	2,26	1,33E-08
SLC12A5	NM_001134771	1,64	0	FURIN	NM_002569	2,19	0
ZNF205	NM_003456	1,64	1,41E-12	IL4R	NM_001257407	2,08	0,00061798
ZNF785	NM_152458	1,63	0,005975541	AKR1C1	NM_001353	2,01	0
RPS20	NM_001146227	1,63	0,029480662	AKR1C1	NM_001353	1,95	0
LENG8	XM_005277062	1,6	0,001737726	APOE	NM_001302688	1,93	0
OBSCN	NM_052843	1,59	0,020220546	CXCL12	NM_199168	1,92	0
GOLGA6A	NM_001038640	1,59	0,012203272	HAS1	NM_001523	1,89	0
ASB5	NM_080874	1,58	1,59E-10	APOC1	NM_001645	1,87	7,16E-06

*FC: Fold change

**Table 2:** Top 15 most down-regulated genes under high glucose condition

Day 1				Day 14			
Gene	RefSeq Accession	FC*	q-value	Gene	RefSeq Accession	FC*	q-value
TMEM67	NM_153704	-2,25	3,92E-06	PF4V1	NM_002620	-4,37	1,84E-194
TOP2A	NM_001067	-1,93	1,55E-41	SLC7A5	NM_003486	-4,09	7,42E-64
SFRP4	NM_003014	-1,82	0,002423432	ANKRD37	NM_181726	-3,68	7,01E-117
VCAM1	NM_001078	-1,81	2,13E-08	TRIB3	NM_021158	-3,65	3,57E-240
GCNT4	NM_016591	-1,8	0,00021802	TCAF2	NM_173678	-3,39	4,69E-27
ATF3	NM_001040619	-1,79	0,003406252	ADM2	NM_024866	-3,33	1,60E-43
ZNF491	NM_152356	-1,78	0,000762658	CHAC1	NM_024111	-3,27	2,48E-35
KRT18	NM_000224	-1,75	0,002937464	FAM46C	NM_017709	-3,22	1,15E-20
RDX	NM_001260492	-1,75	0,000497478	PSAT1	NM_058179	-3,2	6,60E-182
SUPT16H	NM_007192	-1,73	2,62E-06	VLDLR	NM_003383	-3,11	2,87E-23
CDK8	NM_001260	-1,72	1,85E-07	CHAC1	NM_024111	-2,9	2,64E-146
BCO2	NM_031938	-1,7	0,000723735	PPFIA4	XM_006711588	-2,84	1,51E-18
FOS	NM_005252	-1,68	0,001704487	SYT7	NM_001252065	-2,83	2,28E-25
PDK3	NM_005391	-1,67	0,008945477	ASNS	NM_001673	-2,83	1,45E-171
ANLN	NM_018685	-1,67	2,33E-25	EFCAB3	NM_001144933	-2,79	4,46E-14

*FC: Fold change

### Pathway and gene ontology enrichment analysis

**Figure 2:**
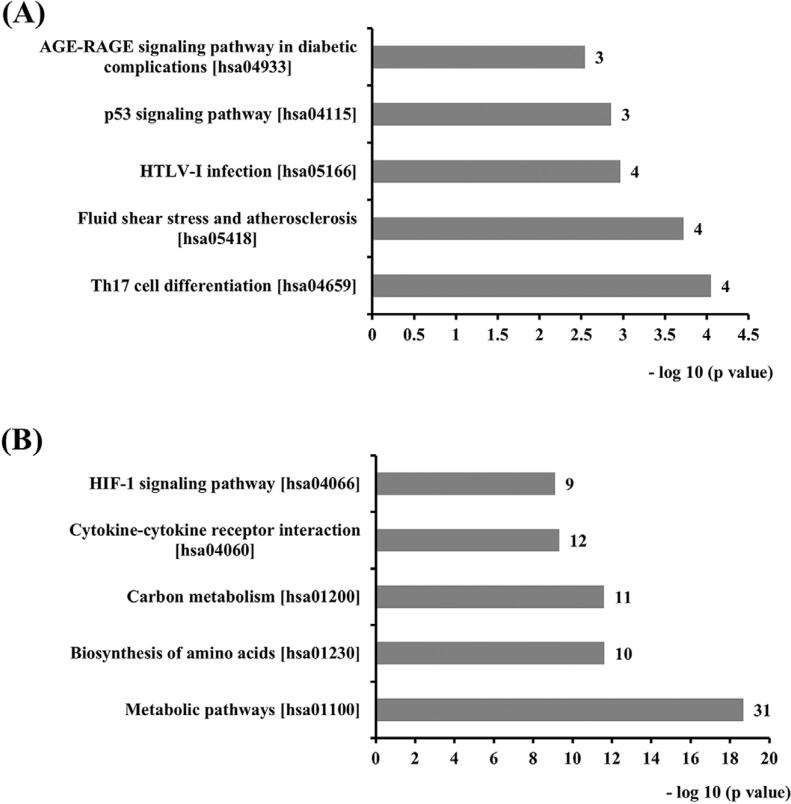
The top 5 KEGG pathways involving DEGs on day 1 (A) and day 14 (B), numbers at the right of each column indicate the number of hit genes in the pathway

**Figure 3:**
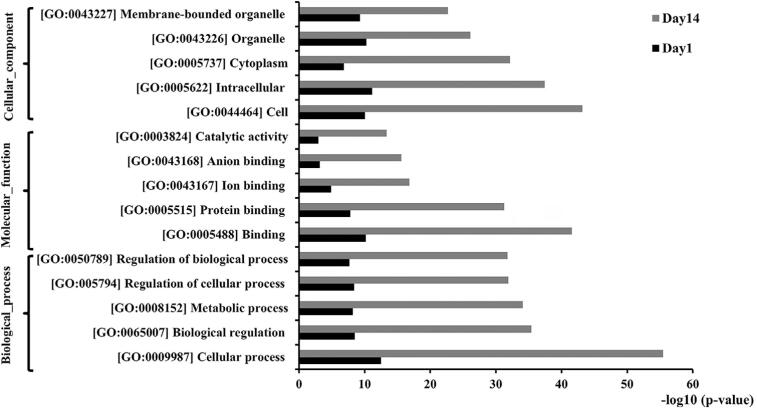
The top 5 most enriched GO terms for each subgroup based on the level of significance, which consisted of cellular component, molecular function and biological process

Kyoto Encyclopedia of Genes and Genomes (KEGG) pathway analysis was done for a better understanding of the pathways involved with DEGs. The top 5 KEGG pathways are depicted in Figure 2. Gene ontology (GO) enrichment analysis for significant genes was done to further investigate the functions of DEGs. The top 5 most enriched GO terms for each subgroup (cellular component, molecular function and biological process) based on the level of significance (log p value) are displayed in Figure 3, and the top 15 most enriched GO terms for each subgroup are shown in Tables S3 and S4.

### RT-qPCR validation

**Figure 4:**
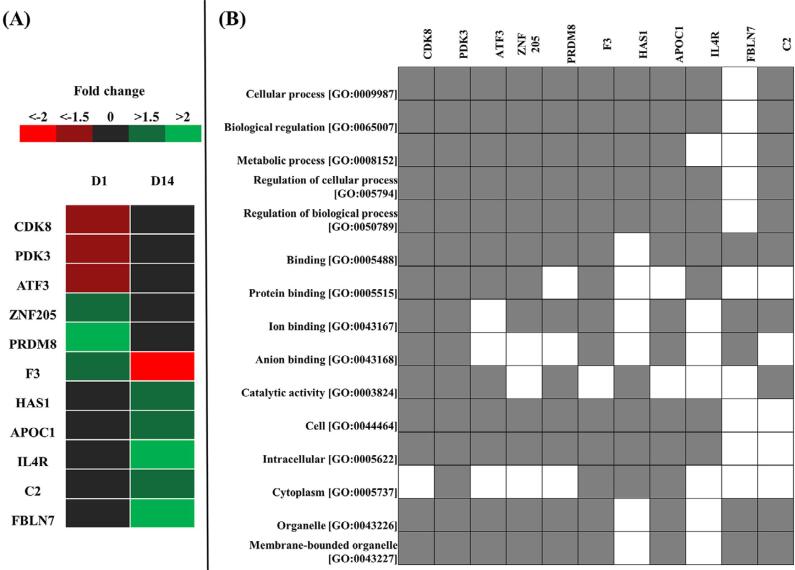
Mapping of selected genes. (A) Heat map of the selected genes displaying fold change. (B) Map of selected genes that were involved in the top 5 most enriched GO terms. Day 1 (D1) and Day 14 (D14)

The DEGs compared between high-glucose and low-glucose conditions on day 1 and day 14 were further selected for qPCR validation based on the selection criteria that the genes were included in the top 15 most significantly up- or down-regulated genes (Table 1) and the genes must also be involved in the top 5 most enriched GO terms (Figure 3). Eventually, 6 of the genes on day 1 and 6 of the genes on day 14 were selected for further validation. Heat maps of the selected genes based on their expression levels are illustrated in Figure 4A, and mapping of the selected genes according to their involvement in the top 5 most enriched GO terms is shown in Figure 4B.

**Figure 5:**
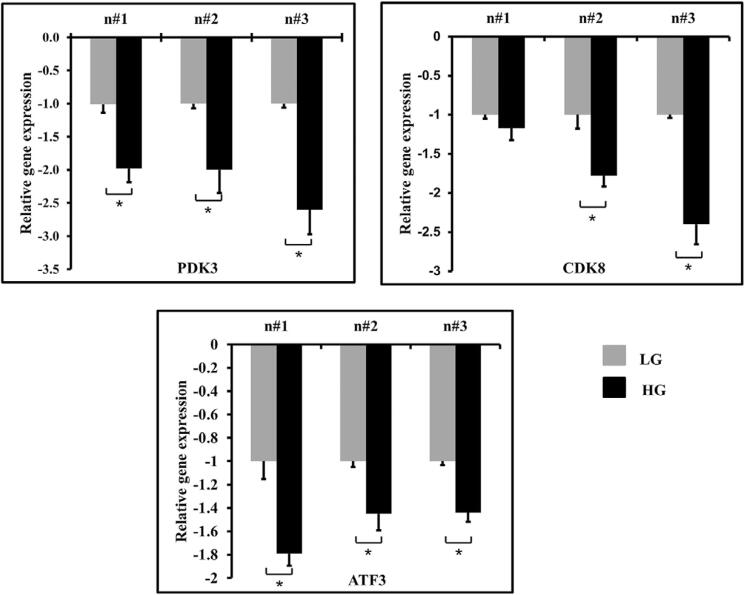
RT-qPCR validation of selected genes on day 1. The mRNA expression was validated from dental pulp cells obtained from 3 individuals: n#1 (donor number 1), n#2 (donor number 2), n#3 (donor number 3). Low glucose (LG) and High glucose (HG). * indicates a statistically significant difference between groups (p< 0.05)

RT-qPCR was used as a validation method for confirming the obtained results. On day 1, 3 of the 6 selected genes, namely pyruvate dehydrogenase kinase 3 (PDK3), cyclin-dependent kinase 8 (CDK8) and activation transcription factor 3 (ATF3), exhibited a similar expression pattern with the microarray results. The mRNA obtained from all 3 donors exhibited a significantly lower expression of PDK3 and ATF3 in the high-glucose condition. The expression pattern of CDK8 in all 3 donors was also found to be down-regulated in the high-glucose condition. However, only 2 of the 3 samples were found to have statistically significant differences, as shown in Figure 5.

**Figure 6:**
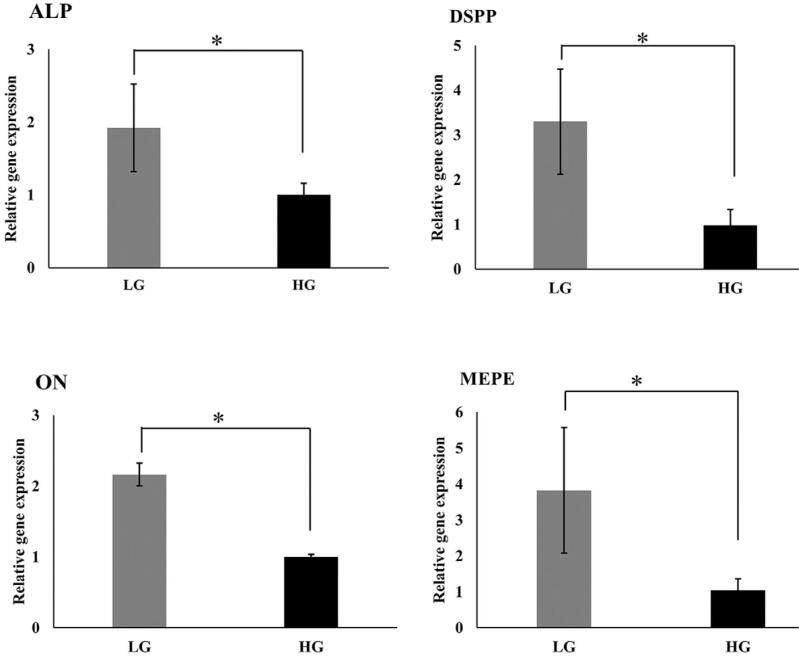
RT-qPCR validation of selected genes on day 14. The mRNA expression was validated from dental pulp cells obtained from 3 individuals: n#1 (donor number 1), n#2 (donor number 2), n#3 (donor number 3). Low glucose (LG) and High glucose (HG). * indicates a statistically significant difference between groups (p< 0.05)

On day 14, 4 of the 6 selected genes, namely fibulin-7 (Fbln-7), hyaluronan synthase (HAS1), interleukin-4 receptor (IL-4R) and apolipoprotein C1 (ApoC1), exhibited a parallel pattern with the results obtained from microarray data (Figure 6). The expressions of the genes Fbln-7*,* IL-4R and ApoC1 were significantly up-regulated in the high-glucose condition in all the mRNA samples obtained from the 3 donors. However, 2 of the 3 samples exhibited a significantly higher expression of HAS1 when compared to the high- and low-glucose conditions, as depicted in Figure 6.

**Figure 7:**
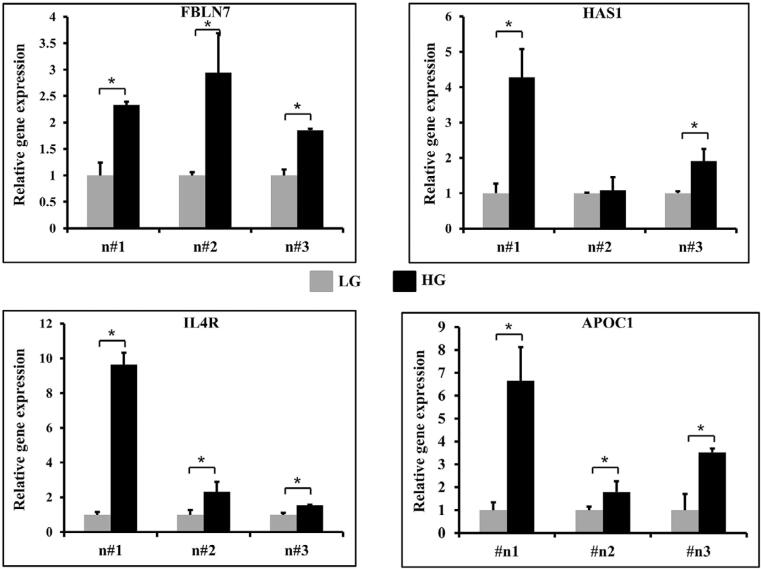
RT-qPCR validation of mineralization-related genes on day 14. Alkaline phosphatase (ALP), dentin sialophosphoprotein (DSPP), matrix extracellular phosphoglycoprotein (MEPE) and osteonectin (ON), Low glucose (LG) and High glucose (HG). * indicates a statistically significant difference between groups (p<0.05)

Mineralization-related genes were validated on day 14. The results showed that the high-glucose condition significantly reduced the expression of alkaline phosphatase (ALP), dentin sialophosphoprotein (DSPP), matrix extracellular phosphoglycoprotein (MEPE) and osteonectin (ON) as shown in Figure 7.

### Formation of mineralized nodules

**Figure 8:**
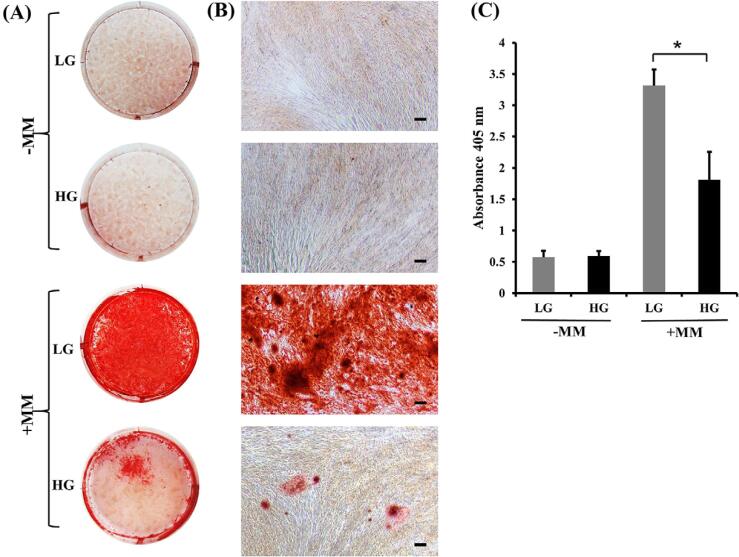
Mineralization of hDPCs. Staining of the alizarin red S–calcium complex; (A) overview, (B) original magnification 40× (scale bars = 100 μm) and (C) quantitation of mineral deposition. Mineralizing medium (MM), Low glucose (LG) and High glucose (HG). * indicates a statistically significant difference between groups (p<0.05).

Alizarin red S staining done for visualizing the calcium complex demonstrated lower alizarin red staining under high-glucose than under low-glucose condition (Figure 8A and B). In addition, quantification of the alizarin red S–calcium complex revealed a significantly higher (alizarin red S) concentration under low-glucose than under high-glucose condition (Figure 8C). These results clearly revealed an inhibitory effect of the high-glucose environment on hDPC mineralization.

## Discussion

**Figure S2:**
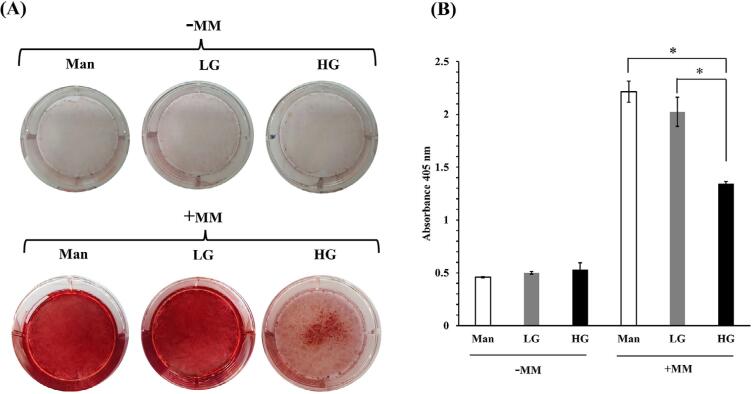
- Mineralization of hDPCs. Staining of the alizarin red S–calcium complex; (A) Overview, (B) quantitation of mineral deposition. Mineralizing medium (MM), Low glucose (LG), 25 mM Mannitol (Man), and High glucose (HG). * indicates a statistically significant difference between groups (p< 0.05)

Hyperglycemia, a major feature of diabetes mellitus, is also considered one of the key factors contributing to its several complications. In this study, we used a glucose concentration of 5.5 mM (100 mg/dl) as an equivalent to normal blood glucose level, whereas a glucose concentration of 25 mM (450 mg/dl) was used to reflect a high blood glucose level (hyperglycemia). Other study has also reported significant changes osteoblast function when treated with 25 mM glucose.^13^ Furthermore, another study used 450 mg/dl glucose concentration to represent hyperglycemic condition in an animal wound healing model.^14^ The results of our study confirmed the negative effect of high levels of glucose. We observed that the high-glucose environment inhibited the mineralization of hDPCs. Lower expression of mineralization-related genes was also observed in high glucose condition. When using 25 mM mannitol as osmotic control, the results showed that the inhibitory effect of high glucose on hDPCs differentiation and mineralization was solely from the direct influence of high glucose and not from osmotic pressure, as shown in Figure S2. A previous study also confirmed that an inhibitory effect of high glucose on hDPCs differentiation was not a result of change in osmolarity.^15^ In this study we investigated the gene expression profile during cell mineralization to identify the genes involved in the effect of glucose on hDPCs mineralization.

Microarray was used as a tool to analyze the gene expression profiling in our study because it has been found to be a high-throughput technique.^10^ This technique permits the investigation and comparison of gene expression patterns in different environments and further identifies the genes that are differently expressed under different conditions. To our knowledge, our study is the first to investigate the gene expression profile of hDPCs during mineralization under the influence of high-glucose condition. The mRNA samples used for microarray analysis were collected on day 1 and day 14 as a representative of early and late responses, respectively. Several studies have suggested that after 1 day of induction, the cells are in the lineage acquisition and early proliferative phase.^16^^,^^17^ However, a period of 14 days after induction would indicate the transition to the mineralization phase.^17^ In addition, the results of our recent study have shown that mineralization began occurring on day 14.^15^ In this study, we used pooled RNA for microarray analysis to reduce the effect of individual heterogeneity.

Results of GO and functional enrichment analysis suggested that DEGs may be involved in the cellular response to stress, inflammation and metabolic pathways, including glucose and carbon metabolism, implying that these pathways may be responsible for the alteration of dental pulp response to a high-glucose environment. Furthermore, a high glucose level may regulate its effect through cellular, biological and metabolic processes of the cells. We then further selected those genes that were included among the top 15 most significantly up- or down-regulated genes and also involved in the top 5 most enriched GO terms of each subgroup for validation. Consequently, 6 genes on day 1 and 6 genes on day 14 were chosen for further validation. We observed that PDK3, CDK8, ATF3, Fbln-7, HAS1, IL-4R and ApoC1 exhibited similar patterns to those of the microarray analysis. Therefore, these genes are good candidates for further investigation and may have important functions in the process of pulp regeneration or pulp inflammation.

The pyruvate dehydrogenase complex (PDC) plays a vital role in glucose metabolism. It provides the link between glycolysis and mitochondrial oxidative phosphorylation by catalyzing the conversion of pyruvate into acetyl Co-A for use in mitochondrial respiration. In general, cells depend on oxidative phosphorylation as a primary pathway to generate energy. However, excessive oxidative phosphorylation may generate harmful ROS production that can cause damage to the cells.^18^ PDK3 is a member of pyruvate dehydrogenase kinase (PDK1-4) isozymes that suppress or inactivate the action of PDC. In tumor cells, a higher expression of PDKs has been shown to decrease ROS production, which in turn promoted the survival and metastasis of cells.^19^ In addition, an increase in cellular oxidative stress has been reported when PDK3 was inhibited in cancer cells.^20^ Because PDK3 has a protective effect on cells against ROS and cellular damage, lower expression of PDK3 under the high-glucose condition could increase ROS production in the dental pulp and cause damage to the cells, which could result in impaired healing and interfere with the ability of pulp cells to form dentin.

CDK8 is a member of the cyclin-dependent protein kinase family. CDK8, along with cyclin C, is a component of the highly conserved module that associates with the RNA polymerase II mediator complex and acts as a positive or negative regulator of transcription.^21^ The cyclin C–Cdk8 complex has been shown to repress the transcription of several stress-responsive genes.^22^ To relieve this repression, Cooper, et al.^23^ (2012) performed an experiment on yeast cells by exposing them to oxidative stress that led to the destruction of the cyclin C–Cdk8 complex, thereby resulting in the loss of cell wall integrity. Furthermore, Chang, Kang and Doering^24^ (2019) reported that deletion of CDK8 resulted in fragmented mitochondrial morphology, susceptibility to oxidative stress and decreased virulence of *Cryptococcus neoformans*. The cyclin C–Cdk8 complex has been found to repress the stress-responsive transcription factors, leading to the inhibition of the stress-inducible pathway; therefore, down-regulation of CDK8 using a high glucose level may activate the stress-responsive mechanisms in pulp cells leading to inflammation and delay of regenerative process in the dental pulp.

ATF3 is known as a stress-inducible gene. It is a member of the ATF/CREB family of transcription factors. ATF3 acts as a transcription activator or repressor depending on its homology.^25^ The role of ATF3 in hard tissue formation remains mostly unknown. However, the overexpression of ATF3 has been reported to inhibit the transcription of alkaline phosphatase.^26^ ATF3 also acts as a negative regulator of the inflammatory response by repressing the expression of pro-inflammatory cytokines.^27^ The down-regulation of ATF3 in high-glucose environment could indicate pulpal inflammation that may delay the process of dentin formation.

Fbln-7 is a member of the fibulin family. It has been reported that Fbln-7 is expressed in pre-odontoblasts and odontoblasts during tooth development.^28^ It was also found in predentin and along the dentinal tubules and was suggested to play a role in odontoblast differentiation and dentin matrix mineralization.^28^ Interestingly, after 14 days of odontogenic induction in our study, we found a higher expression of Fbln-7 in cells that were exposed to high glucose. This may indicate a delay in the process of dentinogenesis, thereby leading to an impairment in the healing and regenerative process of dental pulp under high-glucose environment.

Hyaluronan synthase (HAS) is an enzyme that polymerizes hyaluronan (HA), a glycosaminoglycan found in the dentin extracellular matrix and was highly expressed in odontoblasts and ameloblasts in the early stage of hard tissue formation. The expression of HAS was gradually decreased with the development of teeth.^29^ In addition, Yang, et al.^30^ (2016) identified three isoforms of HAS (HAS1, 2, 3) in dental mesenchymal cells and odontoblasts of embryonic mouse molars and reported that HAS1 expression had decreased in more developing tooth germ. HA and HAS play a role during the early phase of dental hard tissue mineralization. The up-regulation of HAS1 under the high-glucose condition after 14 days of odontogenic induction suggests a delay in the process of mineral dentin formation. Therefore, higher expression of HAS1 may be involved in the impairment of wound healing and regenerative processes in the dental pulp.

ApoC1 is commonly known as a protein that plays a role in lipid metabolism and transport.^31^ The role of ApoC1 in mineral matrix formation remains unknown. It has been found to be associated with diabetes and inflammatory response. One study reported that ApoC1 transgenic mice were protected from obesity and insulin resistance.^32^ High-glucose condition was shown to increase ApoC1 expression in retinal ganglion cells. Furthermore, knock down of ApoC1 inhibited the expression of inflammatory cytokines and cell apoptosis.^33^ APOC1 was also found to enhance the lipopolysaccharide-induced inflammatory response.^34^ As APOC1 is associated with inflammatory response and diabetes, the above-mentioned findings could occur in the dental pulp leading to interference in the regenerative process.

Interleukin-4 (IL-4) is a cytokine that binds to IL-4R. The IL-4/IL-4R axis has been reported to play a role in immunity and inflammation.^35^ Although IL-4 is commonly produced by T cells, mast cells and basophils,^35^ it is also expressed by dental pulp and osteoblast-like cells. High levels of IL-4 and other pro- and anti-inflammatory cytokines have been detected in the dental pulp of carious teeth.^36^ Furthermore, elevated levels of IL-4 have been reported in patients with type I diabetes with hyperglycemia.^37^ However, the role of IL4R expression in dental pulp cells under high glucose is inconclusive.

A suitable further study may determine the key protein involved in the pulp regeneration process. Manipulation of these selected genes may provide a novel insight into the pulp regeneration process in the infected pulp or in dental pulpitis in patients with uncontrolled diabetes and could further increase the rate of success of dental treatment in the future.

Unfortunately, we did not observe any significant changes in the expression level of genes involved in the intracellular glucose metabolism namely; glucose transporter 1. Moreover, dentin formation namely; nestin and collagen 1, and oxidative metabolism-related enzyme namely; superoxide dismutase also showed no significant different in high glucose condition.

We did not observe any significant changes in the expression level of genes involved in the intracellular glucose metabolism namely; glucose transporter 1. Moreover, dentin formation namely; nestin and collagen 1, and oxidative metabolism-related enzyme namely; superoxide dismutase also showed no significant difference that considered to lack of high glucose condition effect. This may be due to the phase of cell differentiation.^16^^,^^17^

The limitation of this study is that it is an *in vitro* experiment, which cultured dental pulp-derived cell in a two-dimension culture system under high glucose concentration which is the important characteristic of diabetes. However, the effect of signal transduction pathway, vascularization and hormonal changes were not presented in this study. The experiment could only control the hyperglycemic factor which may not represent the entire condition in diabetic patients. Another limitation is that osmotic pressure in the medium possibly affected gene expression without affecting *in vitro* mineralization.

In this study, we controlled the quantity of cell culture by normalizing RNA concentration. However, number of cells should clarify further the possible influence of cell survival/apoptosis on experimental conditions.

The study used pooled RNA to reduce individual heterogeneity, which may not be able to rule out the effect of dominant donor; however, the results of CDK8 and HAS1 from all the 3 samples exhibited a similar trend. In addition to the selected genes, we found other interesting genes that were up-regulated in the high-glucose environment, such as apolipoprotein E, tenascin C and claudin 23. These genes have been reported to be associated with osteoblast functions, hard tissue formation, tissue healing and diabetes.^38^^-^^40^ Therefore, it is necessary to further investigate the role of these genes in pulp cells under hyperglycemic condition.

High glucose level has shown to inhibit the mineralization of dental pulp cells. The results of this study provide basic knowledge about the effect of high glucose on gene expression during the odonto- and osteogenic differentiation of hDPCs. This provides the basic knowledge with potential for future dental therapeutic application.

## Conclusion

To our knowledge, this is the first reported gene expression profile of hDPCs during mineralization under high-glucose condition. The high-glucose condition significantly inhibited the odonto- and osteogenic differentiation of hDPCs. DEGs were identified, and interestingly, PDK3, CDK8, ATF3, HAS1, FBLN7, IL4R and APOC1 genes may be involved in the inhibitory effect of glucose on the mineralization of hDPCs.

## Appendix

**Table S1:** -The primer sequences for the selected genes

Genes	Gene Bank No.	Sequences (5’-3’)	Annealing temp (°C)	Product size (bp)
CDK8	NM_001260	F: ACCTCAGACTATCAGCGTTCC	56	153
		R: TTCCGATGCAGCTCAGTACC		
PDK3	NM_005391	F: CCCCTTTGGCTGGATTTGGT	64	197
		R: AGGCGTGGTCTTGTAATGGC		
ATF3	NM_001040619	F: ATCACAAAAGCCGAGGTAGC	60	92
		R: TTGTTTCGGCACTTTGCAGC		
FBLN7	NM_153214	F: CGTCTCCATTCCAGTGTGAGC	66	77
		R: GGAGATGGTCTTGGGCAGAT		
HAS1	NM_001523	F: TATAGGAATAACCTCTTGCAGCAGT	62	143
		R: GACCTGGAGGTGTACTTGGTAG		
IL4R	NM_001257407	F: AACGACCCGGCAGATTTCAG	60	245
		R: CAGACGGCCAGGATGACAAT		
APOC1	NM_001645	F: AGATGCGGGAGTGGTTTTCA	60	180
		R: TTTCTGTAGGATTTTTATTGGTGG		
ZNF205	NM_003456	F:AATGGGCTGTCACTGGGCTTT	64	216
		R:GACTCTCCCTGCCCTGGTTC		
PRDM8	NM_020226	F: GTATATCTTTCGGGTAGACAC	60	170
		R: ACTCCTCGTCTTTGGCAATC		
F3	NM_001993	F: CTGTTCAAATAAGCACTAAGTCAGG	60	233
		R: TGTTGGCTGTCCGAGGTTTG		
C2	NM_001282458	F: GTCGCCTTGAATGGGAGCAA	60	242
		R: CTCACCAGACCCACCTGAAAA		
UBC	NM_021009	F: AAAGTAGTCCCTTCTCGGCG	60	175
		R: CACGAAGATCTGCATTGTCAAG		
MEPE	NM_001184694.2	F:GGCAGCTATCCACACCAGA	62	119
		R:CTGTGGTTGAAATGTTGGTGC		
DSPP	NM_014208.3	F: CAGGACCATGGGAAAGAAGATG	60	284
		R: TCTATTCCCTTATCTTGGCTCTTCC		
ON	NM_003118.3	F: CTGGACTACATCGGGCCTTG	60	174
		R: ATGGATCTTCTTCACCCGCA		
ALP	NM_001177520.2	F: CCTATTGGGTCTCTTCGAGCC	60	269
		R: CCACGGTCAGAGTGTCTTCC		

**Table S2:** - The number of detected probes

Samples	number of probs
Day1_LG (1)	26287
Day1_LG (2)	26635
Day1_HG (1)	26515
Day1_HG (2)	27423
Day14_LG (1)	28328
Day14_LG (2)	26183
Day14_HG (1)	26791
Day14_HG (2)	26562

**Table S3:** - The top 15 most enriched GO terms on day 1

Subgroup	GOID	Term	count	P-Value	Bonferroni	FDR
biological_process	GO:0009987	cellular process	66	3,10401E-13	9,05441E-10	4,5272E-10
biological_process	GO:0071840	cellular component organization or biogenesis	36	1,50684E-09	4,39546E-06	3,13961E-07
biological_process	GO:0065007	biological regulation	50	3,26928E-09	9,5365E-06	6,35767E-07
biological_process	GO:0050794	regulation of cellular process	47	3,86446E-09	1,12726E-05	7,0454E-07
biological_process	GO:0008152	metabolic process	51	6,63755E-09	1,93617E-05	1,13893E-06
biological_process	GO:0050789	regulation of biological process	47	2,08526E-08	6,0827E-05	3,20142E-06
biological_process	GO:0071704	organic substance metabolic process	46	3,29156E-08	9,60148E-05	4,80074E-06
biological_process	GO:0009058	biosynthetic process	33	7,5081E-08	0,000219011	9,65284E-06
biological_process	GO:0044238	primary metabolic process	44	1,09913E-07	0,000320616	1,3359E-05
biological_process	GO:0044237	cellular metabolic process	44	1,27525E-07	0,000371989	1,48796E-05
biological_process	GO:0032502	developmental process	31	2,84198E-07	0,000829006	2,96073E-05
biological_process	GO:0044260	cellular macromolecule metabolic process	38	3,81571E-07	0,001113042	3,71014E-05
biological_process	GO:0043170	macromolecule metabolic process	40	4,1522E-07	0,001211197	3,90709E-05
biological_process	GO:0010467	gene expression	29	4,84121E-07	0,001412182	4,27934E-05
biological_process	GO:0051179	localization	30	5,07577E-07	0,001480602	4,35471E-05
cellular_component	GO:0043229	intracellular organelle	56	3,68031E-12	1,07355E-08	2,68387E-09
cellular_component	GO:0005622	intracellular	61	6,96283E-12	2,03106E-08	4,06212E-09
cellular_component	GO:0043226	organelle	57	5,30532E-11	1,54756E-07	2,2108E-08
cellular_component	GO:0005623	cell	64	8,726E-11	2,54537E-07	2,54537E-08
cellular_component	GO:0043231	intracellular membrane-bounded organelle	50	4,31189E-10	1,25778E-06	1,14343E-07
cellular_component	GO:0043227	membrane-bounded organelle	53	5,02714E-10	1,46642E-06	1,22201E-07
cellular_component	GO:0005737	cytoplasm	45	1,47235E-07	0,000429485	1,65186E-05
cellular_component	GO:0044446	intracellular organelle part	37	3,46942E-07	0,001012031	3,48976E-05
cellular_component	GO:0043232	intracellular non-membrane-bounded organelle	21	2,60577E-05	0,076010212	0,000894238
cellular_component	GO:0005634	nucleus	29	7,32074E-05	0,213546014	0,002016064
cellular_component	GO:0005856	cytoskeleton	14	0,000108623	0,316853797	0,002804016
cellular_component	GO:0043233	organelle lumen	21	0,000130832	0,381636244	0,003289968
cellular_component	GO:0043234	protein complex	21	0,000138753	0,404742086	0,003408642
cellular_component	GO:0031974	membrane-enclosed lumen	21	0,000154884	0,451796164	0,003733853
cellular_component	GO:0070013	intracellular organelle lumen	20	0,000316774	0,924029544	0,00650725
molecular_function	GO:0005488	binding	60	6,3878E-11	1,86332E-07	2,32915E-08
molecular_function	GO:0005515	protein binding	47	1,50265E-08	4,38324E-05	2,43514E-06
molecular_function	GO:1901363	heterocyclic compound binding	31	4,70708E-07	0,001373055	4,27934E-05
molecular_function	GO:0097159	organic cyclic compound binding	31	6,22982E-07	0,001817237	4,98365E-05
molecular_function	GO:0043167	ion binding	29	1,31594E-05	0,03838605	0,000527314
molecular_function	GO:0003676	nucleic acid binding	22	2,1736E-05	0,063403971	0,000782765
molecular_function	GO:0005524	ATP binding	12	0,00016261	0,474332203	0,00382487
molecular_function	GO:0032559	adenyl ribonucleotide binding	12	0,000196522	0,573253755	0,004375983
molecular_function	GO:0030554	adenyl nucleotide binding	12	0,000205592	0,599712592	0,004509117
molecular_function	GO:0044822	poly(A) RNA binding	10	0,000500158	1	0,009352316
molecular_function	GO:0000166	nucleotide binding	14	0,000624475	1	0,011246245
molecular_function	GO:1901265	nucleoside phosphate binding	14	0,000624475	1	0,011246245
molecular_function	GO:0043168	anion binding	15	0,000652926	1	0,011613322
molecular_function	GO:0035639	purine ribonucleoside triphosphate binding	12	0,000878427	1	0,014897505
molecular_function	GO:0032550	purine ribonucleoside binding	12	0,000906221	1	0,015280041

**Table S4:** - The top 15 most enriched GO terms on day 14

Subgroup	GOID	Term	count	PValue	Bonferroni	FDR
biological_process	GO:0009987	cellular process	221	3,25287E-56	1,76176E-52	1,76176E-52
biological_process	GO:0065007	biological regulation	166	3,94018E-36	2,134E-32	2,37111E-33
biological_process	GO:0008152	metabolic process	168	7,74001E-35	4,19199E-31	4,19199E-32
biological_process	GO:0050794	regulation of cellular process	152	1,33061E-32	7,20659E-29	6,00549E-30
biological_process	GO:0050789	regulation of biological process	156	1,70004E-32	9,20742E-29	7,08263E-30
biological_process	GO:0050896	response to stimulus	135	7,07358E-32	3,83105E-28	2,55403E-29
biological_process	GO:0044710	single-organism metabolic process	106	2,07038E-30	1,12132E-26	7,00825E-28
biological_process	GO:0071704	organic substance metabolic process	148	7,65906E-29	4,14815E-25	2,44009E-26
biological_process	GO:0044237	cellular metabolic process	145	2,20891E-28	1,19635E-24	6,64638E-26
biological_process	GO:0006950	response to stress	88	3,02349E-28	1,63752E-24	8,61854E-26
biological_process	GO:0032501	multicellular organismal process	116	2,79272E-27	1,51254E-23	7,56269E-25
biological_process	GO:0044238	primary metabolic process	142	5,0097E-27	2,71325E-23	1,29203E-24
biological_process	GO:0044707	single-multicellular organism process	113	1,06769E-26	5,78259E-23	2,51417E-24
biological_process	GO:0007154	cell communication	106	8,82043E-25	4,77715E-21	1,91086E-22
biological_process	GO:0051716	cellular response to stimulus	111	1,86327E-24	1,00915E-20	3,88134E-22
cellular_component	GO:0005623	cell	211	6,65112E-44	3,60224E-40	7,20449E-41
cellular_component	GO:0005622	intracellular	188	3,91823E-38	2,12211E-34	3,03159E-35
cellular_component	GO:0005737	cytoplasm	155	6,97349E-33	3,77684E-29	3,43349E-30
cellular_component	GO:0043226	organelle	161	7,58506E-27	4,10807E-23	1,8673E-24
cellular_component	GO:0043227	membrane-bounded organelle	148	1,995E-23	1,08049E-19	3,27422E-21
cellular_component	GO:0005576	extracellular region	85	1,09836E-22	5,94869E-19	1,56545E-20
cellular_component	GO:0043229	intracellular organelle	138	4,82936E-19	2,61558E-15	5,44913E-17
cellular_component	GO:0005615	extracellular space	43	8,0628E-19	4,36681E-15	8,73363E-17
cellular_component	GO:0016020	membrane	116	2,12396E-18	1,15033E-14	2,25556E-16
cellular_component	GO:0043231	intracellular membrane-bounded organelle	127	1,97265E-17	1,06839E-13	1,72321E-15
cellular_component	GO:0005829	cytosol	57	1,06325E-13	5,75854E-10	6,12611E-12
cellular_component	GO:0031224	intrinsic component of membrane	74	2,53029E-12	1,37041E-08	1,18138E-10
cellular_component	GO:0071944	cell periphery	70	8,88962E-12	4,81462E-08	3,91433E-10
cellular_component	GO:0005886	plasma membrane	68	2,50724E-11	1,35792E-07	9,84E-10
cellular_component	GO:0016021	integral component of membrane	70	5,50786E-11	2,98306E-07	2,02929E-09
molecular_function	GO:0005488	binding	196	2,68925E-42	1,4565E-38	2,4275E-39
molecular_function	GO:0005515	protein binding	154	5,58087E-32	3,0226E-28	2,159E-29
molecular_function	GO:0043167	ion binding	91	1,49172E-17	8,07914E-14	1,33603E-15
molecular_function	GO:0043168	anion binding	56	2,48291E-16	1,34475E-12	1,92106E-14
molecular_function	GO:0003824	catalytic activity	80	4,37976E-14	2,37208E-10	2,60668E-12
molecular_function	GO:0036094	small molecule binding	48	9,37547E-12	5,07776E-08	4,09496E-10
molecular_function	GO:0043169	cation binding	60	1,16729E-10	6,32202E-07	3,97611E-09
molecular_function	GO:0097159	organic cyclic compound binding	72	2,57026E-09	1,39205E-05	6,89135E-08
molecular_function	GO:0097367	carbohydrate derivative binding	39	3,26537E-09	1,76852E-05	8,54359E-08
molecular_function	GO:1901363	heterocyclic compound binding	70	7,16567E-09	3,88093E-05	1,78024E-07
molecular_function	GO:0046872	metal ion binding	55	7,6435E-09	4,13972E-05	1,87318E-07
molecular_function	GO:0005125	cytokine activity	13	9,89574E-09	5,35953E-05	2,38201E-07
molecular_function	GO:0046983	protein dimerization activity	26	1,51409E-08	8,20032E-05	3,53462E-07
molecular_function	GO:0015175	neutral amino acid transmembrane transporter activity	7	5,85648E-08	0,000317187	1,21064E-06
molecular_function	GO:0005102	receptor binding	28	6,507E-08	0,000352419	1,32988E-06

## References

[1] 1 - American Diabetes Association. Classification and diagnosis of diabetes: standards of medical care in diabetes. Diabetes Care. 2020;43(Suppl 1):s14-s31. doi: 10.2337/dc20-S002 10.2337/dc20-S002 31862745

[2] 2 - Catanzaro O, Dziubecki D, Lauria LC, Ceron CM, Rodriguez RR. Diabetes and its effects on dental pulp. J Oral Sci. 2006;48(4):195-9. doi: 10.2334/josnusd.48.195 10.2334/josnusd.48.195 17220616

[3] 3 - Leite MF, Ganzerla E, Marques MM, Nicolau J. Diabetes induces metabolic alterations in dental pulp. J Endod. 2008;34(10):1211-4. doi: 10.1016/j.joen.2008.07.010 10.1016/j.joen.2008.07.010 18793922

[4] 4 - Kodama Y, Matsuura M, Sano T, Nakahara Y, Ozaki K, Narama I, et al. Diabetes enhances dental caries and apical periodontitis in caries-susceptible WBN/KobSlc rats. Comp Med. 2011;61(1):53-9. PMC3060429 21819682

[5] 5 - Yeh CK, Harris SE, Mohan S, Horn D, Fajardo R, Chun YH, et al. Hyperglycemia and xerostomia are key determinants of tooth decay in type 1 diabetic mice. Lab Invest. 2012;92(6):868-82. doi: 10.1038/labinvest.2012.60 10.1038/labinvest.2012.60 PMC4513945 22449801

[6] 6 - Yan LJ. Pathogenesis of chronic hyperglycemia: from reductive stress to oxidative stress. J Diabetes Res. 2014;2014:137919. doi: 10.1155/2014/137919 10.1155/2014/137919 PMC4082845 25019091

[7] 7 - Kato H, Taguchi Y, Tominaga K, Kimura D, Yamawaki I, Noguchi M, et al. High glucose concentrations suppress the proliferation of human periodontal ligament stem cells and their differentiation into osteoblasts. J Periodontol. 2016;87(4):e44-51. doi: 10.1902/jop.2015.150474 10.1902/jop.2015.150474 26537370

[8] 8 - Shah D, Lynd T, Ho D, Chen J, Vines J, Jung HD, et al. Pulp-Dentin tissue healing response: a discussion of current biomedical approaches. J Clin Med. 2020;5;9(2):434. doi: 10.3390/jcm9020434 10.3390/jcm9020434 PMC7074340 32033375

[9] 9 - Garber SE, Shabahang S, Escher AP, Torabinejad M. The effect of hyperglycemia on pulpal healing in rats. J Endod. 2009;35(1):60-2. doi: 10.1016/j.joen.2008.09.010 10.1016/j.joen.2008.09.010 19084126

[10] 10 - Tarca AL, Romero R, Draghici S. Analysis of microarray experiments of gene expression profiling. Am J Obstet Gynecol. 2006;195(2):373-88. doi: 10.1016/j.ajog.2006.07.001 10.1016/j.ajog.2006.07.001 PMC2435252 16890548

[11] 11 - Gronthos S, Arthur A, Bartold PM, Shi S. A method to isolate and culture expand human dental pulp stem cells. Methods Mol Biol. 2011;698:107-21. doi: 10.1007/978-1-60761-999-4_9 10.1007/978-1-60761-999-4_9 21431514

[12] 12 - Gregory CA, Gunn WG, Peister A, Prockop DJ. An Alizarin red-based assay of mineralization by adherent cells in culture: comparison with cetylpyridinium chloride extraction. Anal Biochem. 2004;329(1):77-84. doi: 10.1016/j.ab.2004.02.002 10.1016/j.ab.2004.02.002 15136169

[13] 13 - Miranda C, Giner M, Montoya MJ, Vazquez MA, Miranda MJ, Perez-Cano R. Influence of high glucose and advanced glycation end-products (ages) levels in human osteoblast-like cells gene expression. BMC Musculoskelet Disord. 2016;17:377. doi: 10.1186/s12891-016-1228-z 10.1186/s12891-016-1228-z PMC5007697 27582133

[14] 14 - Mrozikiewicz-Rakowska B, Mieczkowski M, Siwko T, Bujalska-Zadrozny M, Grzela T, Wolinska R, et al. Insulin or metformin for glucose control during wound healing in diabetes? Diabetes. 2018;67(Suppl 1):630-P. doi: 10.2337/db18-630-P 10.2147/DMSO.S296287 PMC8039538 33854349

[15] 15 - Horsophonphong S, Kitkumthorn N, Sritanaudomchai H, Nakornchai S, Surarit R. High glucose affects proliferation, reactive oxygen species and mineralization of human dental pulp cells. Braz Dent J. 2020;31:298-303. doi. 10.1590/0103-6440202003120 10.1590/0103-6440202003120 32667524

[16] 16 - van de Peppel J, Strini T, Tilburg J, Westerhoff H, van Wijnen AJ, van Leeuwen JP. Identification of three early phases of cell-fate determination during osteogenic and adipogenic differentiation by transcription factor dynamics. Stem cell reports. 2017;8(4):947-60. doi: 10.1016/j.stemcr.2017.02.018 10.1016/j.stemcr.2017.02.018 PMC5390132 28344004

[17] 17 - Beck GR Jr, Zerler B, Moran E. Gene array analysis of osteoblast differentiation. Cell Growth Differ. 2001;12(2):61-83. 11243467

[18] 18 - Checa J, Aran JM. Reactive oxygen species: drivers of physiological and pathological processes. J Inflamm Res. 2020;13:1057-73. doi: 10.2147/JIR.S275595 10.2147/JIR.S275595 PMC7719303 33293849

[19] 19 - Jeoung NH. Pyruvate dehydrogenase kinases: therapeutic targets for diabetes and cancers. Diabetes Metab J. 2015;39(3):188-97. doi: 10.4093/dmj.2015.39.3.188 10.4093/dmj.2015.39.3.188 PMC4483603 26124988

[20] 20 - Ren YJ, Wang XH, Ji C, Guan YD, Lu XJ, Liu XR, et al. Silencing of NAC1 expression induces cancer cells oxidative stress in hypoxia and potentiates the therapeutic activity of elesclomol. Front Pharmacol. 2017;8:804. doi: 10.3389/fphar.2017.00804 10.3389/fphar.2017.00804 PMC5681923 29163184

[21] 21 - Nemet J, Jelicic B, Rubelj I, Sopta M. The two faces of Cdk8, a positive/negative regulator of transcription. Biochimie. 2014;97:22-7. doi: 10.1016/j.biochi.2013.10.004 10.1016/j.biochi.2013.10.004 24139904

[22] 22 - Stieg DC, Chang KT, Cooper KF, Strich R. Cyclin C regulated oxidative stress responsive transcriptome in *Mus musculus* embryonic fibroblasts. G3 (Bethesda). 2019;9(6):1901-8. doi: 10.1534/g3.119.400077 10.1534/g3.119.400077 PMC6553531 31036676

[23] 23 - Cooper KF, Scarnati MS, Krasley E, Mallory MJ, Jin C, Law MJ, et al. Oxidative-stress-induced nuclear to cytoplasmic relocalization is required for Not4-dependent cyclin C destruction. J Cell Sci. 2012;125(Pt 4):1015-26. doi: 10.1242/jcs.096479 10.1242/jcs.096479 PMC3311932 22421358

[24] 24 - Chang AL, Kang Y, Doering TL. Cdk8 and Ssn801 Regulate oxidative stress resistance and virulence in *Cryptococcus neoformans*. MBio. 2019;10(1):e02818-18. doi: 10.1128/mBio.02818-18 10.1128/mBio.02818-18 PMC6372802 30755515

[25] 25 - Zhao J, Li X, Guo M, Yu J, Yan C. The common stress responsive transcription factor ATF3 binds genomic sites enriched with p300 and H3K27ac for transcriptional regulation. BMC genomics. 2016;17:335. doi: 10.1186/s12864-016-2664-8 10.1186/s12864-016-2664-8 PMC4857411 27146783

[26] 26 - Park JK, Jang H, Hwang S, Kim EJ, Kim DE, Oh KB, et al. ER stress-inducible ATF3 suppresses BMP2-induced ALP expression and activation in MC3T3-E1 cells Biochem Biophys Res Commun. 2014;443(1):333-8. doi: 10.1016/j.bbrc.2013.11.121 10.1016/j.bbrc.2013.11.121 24315873

[27] 27 - Jadhav K, Zhang Y. Activating transcription factor 3 in immune response and metabolic regulation. Liver Res. 2017;1(2):96-102. doi: 10.1016/j.livres.2017.08.001 10.1016/j.livres.2017.08.001 PMC5724780 29242753

[28] 28 - de Vega S, Iwamoto T, Nakamura T, Hozumi K, McKnight DA, Fisher LW, et al. TM14 is a new member of the fibulin family (fibulin-7) that interacts with extracellular matrix molecules and is active for cell binding. J Biol Chem. 2007;282(42):30878-88. doi: 10.1074/jbc.M705847200 10.1074/jbc.M705847200 17699513

[29] 29 - Felszeghy S, Meszar Z, Prehm P, Modis L. The expression pattern of hyaluronan synthase during human tooth development. Arch Oral Biol. 2005;50(2):175-9. doi: 10.1016/j.archoralbio.2004.10.010 10.1016/j.archoralbio.2004.10.010 15721147

[30] 30 - Yang G, Jiang B, Cai W, Liu S, Zhao S. Hyaluronan and hyaluronan synthases expression and localization in embryonic mouse molars. J Mol Histol. 2016;47(4):413-20. doi: 10.1007/s10735-016-9684-1 10.1007/s10735-016-9684-1 27318667

[31] 31 - Fuior EV, Gafencu AV. Apolipoprotein C1: its pleiotropic effects in lipid metabolism and beyond. Int J Mol Sci. 2019;20(23):5939. doi: 10.3390/ijms20235939 10.3390/ijms20235939 PMC6928722 31779116

[32] 32 - Jong MC, Voshol PJ, Muurling M, Dahlmans VE, Romijn JA, Pijl H, et al. Protection from obesity and insulin resistance in mice overexpressing human apolipoprotein C1. Diabetes. 2001;50(12):2779-85. doi: 10.2337/diabetes.50.12.2779 10.2337/diabetes.50.12.2779 11723061

[33] 33 - Weng Y, Wu W, Wang W, Wan T. Knockdown of APOC1 promotes retinal ganglion cell survival to delay diabetic retinopathy progress. Int J Clin Exp Pathol. 2017;10:3957-63.

[34] 34 - Berbée JF, Coomans CP, Westerterp M, Romijn JA, Havekes LM, Rensen PC. Apolipoprotein CI enhances the biological response to LPS via the CD14/TLR4 pathway by LPS-binding elements in both its N- and C-terminal helix. J Lipid Res. 2010;51(7):1943-52. doi: 10.1194/jlr.M006809 10.1194/jlr.M006809 PMC2882731 20335569

[35] 35 - Junttila IS. Tuning the cytokine responses: an update on Interleukin (IL)-4 and IL-13 receptor complexes. Front Immunol. 2018;9:888. doi: 10.3389/fimmu.2018.00888 10.3389/fimmu.2018.00888 PMC6001902 29930549

[36] 36 - Horst OV, Horst JA, Samudrala R, Dale BA. Caries induced cytokine network in the odontoblast layer of human teeth. BMC Immunol. 2011;12(1):9. doi: 10.1186/1471-2172-12-9 10.1186/1471-2172-12-9 PMC3036664 21261944

[37] 37 - Rosa JS, Flores RL, Oliver SR, Pontello AM, Zaldivar FP, Galassetti PR. Sustained IL-1α, IL-4, and IL-6 elevations following correction of hyperglycemia in children with type 1 diabetes mellitus. Pediatr Diabetes. 2008;9(1):9-16. doi: 10.1111/j.1399-5448.2007.00243.x 10.1111/j.1399-5448.2007.00243.x 18211631

[38] 38 - Alshbool FZ, Mohan S. Differential expression of claudin family members during osteoblast and osteoclast differentiation: Cldn-1 is a novel positive regulator of osteoblastogenesis. PloS one. 2014;9(12):e114357. doi: 10.1371/journal.pone.0114357 10.1371/journal.pone.0114357 PMC4257558 25479235

[39] 39 - Noguchi T, Ebina K, Hirao M, Otsuru S, Guess A, Kawase R, et al. Apolipoprotein E plays crucial roles in maintaining bone mass by promoting osteoblast differentiation via ERK1/2 pathway and by suppressing osteoclast differentiation via c-Fos, NFATc1, and NF-κB pathway. Biochem Biophys Res Commun. 2018;503. doi: 10.1016/j.bbrc.2018.06.055 10.1016/j.bbrc.2018.06.055 29906458

[40] 40 - Li C, Cui Y, Luan J, Zhou X, Li H, Wang H, et al. Tenascin C affects mineralization of SaOS2 osteoblast-like cells through matrix vesicles. Drug Discov Ther. 2016;10(2):82-7. doi: 10.5582/ddt.2016.01009 10.5582/ddt.2016.01009 26961327

